# Oocyte *in vitro* maturation: A sytematic review

**DOI:** 10.4274/tjod.23911

**Published:** 2018-06-21

**Authors:** Şafak Hatırnaz, Barış Ata, Ebru Saynur Hatırnaz, Michael Haim Dahan, Samer Tannus, Justin Tan, Seang Lin Tan

**Affiliations:** 1 Medicana International Hospital, In Vitro Fertilization Center, Samsun, Turkey; 2 Koç University Faculty of Medicine, Department of Obstetrics and Gynecology, In Vitro Fertilization Center, İstanbul, Turkey; 3 Mc Gill University Faculty of Medicine, Department of Obstetrics and Gynecology, Quebec, Canada; 4 Originelle Women and Reproductive Medicine Center, Clinic of Obstetrics and Gynecology, Montreal, Quebec, Canada

**Keywords:** In vitro maturation, clinical applications, laboratory procedure, pregnancy rate, fertility preservation

## Abstract

*In vitro* maturation (IVM) is one of the most controversial aspects of assisted reproductive technology. Although it has been studied extensively, it is still not a conventional treatment option and is accepted as an alternative treatment. However, studies have shown that IVM can be used in almost all areas where in vitro fertilization (IVF) is used and it has a strong place in fertility protection and Ovarian Hyperstimulation syndrome management. The aim of this systematic review was to address all aspects of the current knowledge of IVM treatment together with the evolution of IVM and IVF.

## Introduction

The first in vitro fertilization (IVF) attempts were carried out with immature rabbit oocytes because in vivo matured oocyte retrieval was impossible in the 1930s^(1,2,3,4)^. Edwards.^(5,6,7)^ conducted essential work on human oocyte *in vitro* maturation (IVM) during the 1960s and the first human IVF techniques were based on the use of IVM. IVM is the progenitor of current IVF treatment^(8,9)^. Mature oocyte collection from the preovulatory follicles in normal cycle women only became possible after the introduction of laparoscopy into gynecology practice in the 1970s^(10)^. With the advent of IVF and the successful delivery of Louise Brown, IVF following ovarian stimulation became the norm. Clomiphene citrate (CC), which was first marketed in the 1960s, was the first agent for ovarian stimulation. Later human menopausal gonadotropins (hMGs) were introduced into the IVF industry and either alone or in combination with CC, hMGs became the drug of choice in ovarian stimulation protocols^(11,12,13,14,15)^. Although hMGs increased the number of oocytes and the chance of pregnancy, they brought about Ovarian Hyperstimulation syndrome (OHSS), which could even be fatal for an otherwise healthy young woman^(16)^. This is one of the reasons why IVM regained attention in the 1990s as an alternative. Cha et al.^(17)^ reported the first IVM birth from immature oocytes derived from oocyte donors. The first IVM baby from the mother’s own immature oocytes was in 1994^(18)^. Worldwide, over 5000 IVM babies have since been born^(19)^. Natural-cycle IVF or mild-stimulation protocols have acceptable outcomes and some advantages over traditional IVF cycles, especially in poor responders^(20)^. IVM gained attention among fertility specialists for its safety, repeatability, cost effectiveness, and almost no risk of OHSS together with acceptable clinical pregnancy rates. Though the main indication was patients with polycystic ovaries (PCO), IVM has much wider indications including poor ovarian reserve and repeated IVF failures^(21)^. The term IVM refers to the maturation of the retrieved immature oocytes in the special culture environment. Exogenous gonadotropin stimulation for short courses seems to improve the ultrastructure of the oocytes expected to mature in *in vitro* conditions. In the strict definition, this is not IVM. However, this raises the question of whether a correct definition of IVM or the benefit patients gain from the treatment is more important. The kinetics of oocyte maturation and ultrastructural changes will be discussed in the following text. The nuclear and cytoplasmic maturation in *in vitro* conditions determine the competence of the oocytes and eventually embryo quality and clinical outcomes. Many authors recommend IVM as an alternative to traditional IVF, whereas others refuse to recommend IVM as a treatment option in the modern era^(22,23)^. However, contrary to the American Society for Reproductive Medicine opinion and the publication of De Zeigler, the accumulating data show that IVM is not an alternative, but should be accepted as a potential first-line treatment option^(8,24)^. There are many controversial issues that need to be clarified about IVM. Should we neglect IVM or adopt it as an important treatment option in IVF centers? The aim of this systematic review was to discuss all aspects of IVM in detail, from the definition to clinical outcomes.

### Online literature search

The following keywords were used to search PubMed; *in vitro* oocyte maturation, clinical outcomes, indications, ultrastructural changes, fertility preservation. A total of 2753 papers were seen during PubMed and selective journal searches. A detailed search eliminated most of the papers and 456 papers from both PubMed and hand-searched assisted reproduction technology (ART) journals were re-checked.

### Selection of eligible studies

One hundred forty-three full-text papers and 67 abstracts selected from PubMed and ART journals were selected for this systematic review.

## Discussion

### Terminology and description

Even the definition of human oocyte IVM is controversial. Dahan et al.^(25)^ recently published a paper on the definition of IVM. Variations in treatment protocols, selection of patients, indications other than Polycystic Ovarian syndrome, number of embryos transferred, and cleavage stage embryo or blastocyst transfer are important factors. Thus, a clinical definition of IVM was introduced: The aspiration of small or intermediate-sized follicles for oocyte retrieval in the ovaries carrying follicles less than 13 mm in diameter. Short-course of gonadotropin use needs to be acknowledged but priming during monitorization may lead to the retrieval of mature oocytes together with immature oocytes. Thus, they recommended renaming the procedure as natural- cycle IVF or modified natural-cycle IVF with early triggering, combined with IVM^(25)^. We reported collecting only germinal vesicle (GV) oocytes in follicle-stimulating hormone (FSH)-human chorionic gonadotropin (hCG) primed IVM cycles of 165 patients. The reason is the largest size was less than 10 mm and hCG priming was given on the 8^th^ day of the cycle^(26)^. The rationale for using gonadotropins prior to IVM was to trigger the developmental potential of the immature oocytes. The reason for using gonadotropins prior to IVM is to trigger the developmental potential of the immature oocytes and make them more compatible. Some authors may oppose the above mentioned definitions because they believe that IVM should never be primed with pharmacologic agents^(27)^. Edwards RG.^(28)^ published a paper on the definition of IVF terminology and reported that IVM could be included in the list of definitions by using minimal/mild-stimulation IVM or natural-cycle IVM. There is another important issue that needs to be taken into consideration; one is that immature oocytes retrieved after long-term gonadotropin stimulation and culturing denuded immature oocytes from such conventional stimulated IVF patients is not in the vicinity of IVM and another is truncated IVF where just a single bolus of hCG or gonadotropin agonist is given for triggering without short FSH priming^(29)^. Mixed oocyte generation is seen in both these protocols which are matured *in vitro* and later fertilized^(9)^. Wang et al.^(30)^ published an interesting article in which they studied 591 IVM cycles, 240 cycles of unstimulated IVM for PCO syndrome (PCOS), 153 cycles of IVM converted from stimulated IVF cycles for PCOS, 103 cycles of unstimulated IVM in non-PCOS patients, and 95 cycles of IVM converted from stimulated IVF cycles of non-PCOS cases were compared. They concluded that PCOS and IVM cycles rescued from stimulated cycles have higher implantation rates, better quality embryos, and acceptable clinical pregnancy rates. Also, IVM cycles converted from stimulated IVF cycles have lower abortion rates as compared with others. They also concluded that PCOS cases were more suitable for IVM treatment^(30)^. In an unpublished study 11 cases of stimulated IVF cycles that resisted gonadotropins were converted to rescue IVM and 6 patients had pregnancy and 4 had livebirths (Hatırnaz et al. article in review). The definitions proposed to date remain confusing because a definition should be short and simple, thus both definitions made by Dahan et al.^(25)^ and Coticchio^(27)^ need further evaluation.

### Indications and clinical applications

IVM was first introduced in patients with PCOS and patients who had severe OHSS in their previous IVF treatments but the indications were expanded in recent years and in almost all areas of infertility; IVM can be adapted as an option. Potential indications of IVM are;

-PCOS

-PCO-like ovaries

-Normo-ovulatory patients

-Previous failed IVF attempts

-History of OHSS

-Oocyte maturation problems

-Patients with testicular sperm extraction (microdissection-TESE)

-Emergency oocyte retrieval due to malignancies (estrogen-sensitive tumors)

-Oocyte retrieval from ovarian tissue before vitrification

-Poor responders

-IVM for rescuing IVF cycles

-Resistant Ovary syndrome

-Recurrent implantation failure

-Preimplantation genetic diagnosis (PGD)/preimplantation genetic screening (PGS)

IVM was first introduced into clinical practice as an alternative treatment option in patients with PCOS^(17)^. Trounson et al.^(18)^ reported that immature oocytes derived from IVM cycles retain their potential to grow under* in vitro* conditions and this can be a new therapy for infertile women with PCOS. From that time on, many studies focused on the use of IVM in other indications. Lindenberg^(31)^ reported that IVM was also used in regularly menstruating women, low responders, and in patients with cancer. Papers on IVM before 2009 showed low implantation and pregnancy rates but after the publication of Pak et al.^(32)^, the results were comparable with IVF. PCOS and OHSS are the main indications of IVM; however, IVM may also be used in cases of resistant ovary syndrome and fertility preservation as uncommon indications^(33,34,35)^. Child et al.^(36)^ studied the impact of IVM on PCO, PCOS, and unstimulated normal ovaries and concluded that hCG priming in all three groups had similar high maturation, fertilization, and developmental potential. Seok et al.^(37)^ studied the predictive role of anti-mullerian hormone (AMH) on the selection of IVM in patients with PCOS and concluded that AMH was a valuable factor in predicting clinical outcomes in such patients who preferred IVM as the treatment of choice. Gremeau et al.^(38)^ studied 194 women with PCOS to evaluate the efficacy of IVM instead of conventional IVF and concluded that IVM was safer, simpler, and avoided the risks of OHSS secondary to IVF. Siristatidis et al.^(39)^ reviewed IVM in patients with and without PCOS. In a meta-analysis of 11 studies, 268 patients with PCOS with 328 cycles and 110 patients with PCOS with 110 cycles were compared with 440 patients dendritic cells 1 and it was concluded that IVM was an effective treatment option when offered in subfertile women with PCOS^(39)^. Yoon et al.^(40)^ studied pregnancy outcomes from IVM-derived oocytes in normoovulatory women and found a 17.6% pregnancy rate (9/51 embryo transfers). It was concluded that IVM in normoresponder cases might lead to successful pregnancies though the pregnancy rate was quite low^(40)^. Oocyte collection during the luteal phase opened a new dimension in ART and in patients with cancer wishing to preserve their fertility because luteal phase oocyte pick up is possible and efficient. Demirtas et al.^(41)^ studied three women without male partners who were close to gonadotoxic chemotherapy due to malignancies. IVM oocytes were easily retrieved from luteal phase ovaries in these women and oocytes were vitrified for future use^(41)^. Fadini et al.^(42)^ studied IVM in normoovulatory women and compared IVM with conventional IVF. They found that conventional IVF was superior to IVM in respect of the success rates and IVM could be an alternative intervention for some conditions^(42)^. Fadini et al.^(43)^ also studied predictive factors in IVM and evaluated the role of body mass index, basal FSH and estradiol concentrations, antral follicle counts (AFC), endometrium thickness, and leading follicle size. Estradiol and FSH concentration and AFC were found to be predictive factors in the decision of whether to start IVM, and endometrial thickness and leading follicle were predictive factors for the timing of immature oocyte retrieval^(43)^. Tannus et al.^(44)^ evaluated predictive factors in 159 IVM cases and concluded that duration of infertility, number of immature oocytes, embryo blastomere count, and embryo grade were predictive factors for live birth after IVM in patients with PCOS. Braga et al.^(45)^ studied IVM in stimulated cycles in 440 poor responder patients. Immature oocytes associated with MII oocytes were divided into two groups and rescue spontaneous maturation oocyte-derived embryos were added to* in vivo *matured oocyte derived embryos in poor responder patients. They concluded that adding such embryos in poor responder patients had no impact on clinical outcomes, although it improved the number of embryos transferred and lowered the cancellation rates^(45)^. IVM may be a valuable option in patients who failed in conventional IVF. Gulekli et al.^(46)^ studied 23 women who failed in conventional IVF and were transferred to IVM without ovarian stimulation. Only one pregnancy was obtained and that did not continue to birth, and the authors concluded that IVM might be a useful tool for failed conventional IVF^(46)^. As an uncommon indication, IVM may be an important optional choice in cases with oocyte maturation abnormalities. Hatırnaz and Hatırnaz^(47)^ reported a patient with genuine Empty Follicle syndrome (EFS) who benefited from IVM oocytes retrieved from the patient and matured, but her partner was azoospermic and only a few sperms were derived from the microsurgical TESE (micro-TESE) procedure and one embryo on day 2 was transferred with a negative pregnancy test^(47)^. Hourvitz et al.^(48)^ evaluated 7 patients with seven IVM cycles. Two of them were genuine EFS, one was PCOS with egg factor, two patients had repeated GV oocytes in their retrievals, and two had atretic oocytes. The patients with genuine EFS achieved pregnancy and the course of the other indications was not relevant. IVM may be the first choice in cases of genuine EFS^(48)^. Edwards reviewed new modalities that may replace routine IVF (IVM, natural-cycle IVF, minimal-stimulation IVF) and gave special attention to IVM. The author discussed the huge amount of information collected from the genetics and biochemistry of IVM oocytes and reviewed papers enlightening the future, and suggested that new follicular formation could be achieved from bone marrow or stem cells derived from a drop of blood both in children and adults^(49)^. There is a need for clarification of whether IVM should be evaluated strictly as a laboratory procedure alone or be accepted as part of the IVF treatment protocol. In the following section, types of ovarian stimulation and additional measures to optimize IVM outcomes from clinical site will be discussed.

### Stimulation protocols and treatment modalities in in vitro maturation

The only difference of IVM from conventional IVF is the maturation of the oocytes under *in vitro* conditions. IVM is a laboratory term and obtaining immature oocytes is dependent on certain clinical protocols, monitorization, and timing of oocyte retrieval, which is why IVM per se is not a treatment protocol, it is the laboratory part of a stimulation protocol or a stimulation cycle. Types of stimulations are listed below:

- Unstimulated IVM cycles without hCG priming

- FSH priming IVM cycles (75 IU/day for 3 days. Start at day 3)

- hCG priming IVM cycles (10.000 IU-20.000 IU IM when the endometrium reaches 8 mm)

- FSH and hCG priming IVM cycles (the combination of above protocols)

- Cycle independent IVM in cancer patients (random start or letrozole use)

- IVM cycles converted from conventional IVF (rescue procedure)

- Aromatase inhibitor use for ovarian stimulation in IVM (letrozole 2.5 mg twice daily start at day 3 for five days)

- Estrogen-suppressed in IVM (estradiol valearate started on day 3 of the cycle)

IVM is not something that promotes the initiation of processes that activate quiescent human oocytes. A fully-grown oocyte meiotic resumption is triggered by luteinizing hormone (LH) or by hCG administration before oocyte retrieval. Oocytes are covered by a thick layer of glycoprotein secreted by the oocyte itself called the zona pellucida. The zona is covered by specialized granulosa cells named corona cells, which make the cumulus oocyte complex (COCs) for the good nourishment of the oocytes. Oocyte maturation means the nuclear and cytoplasmic maturation processes, which should not necessarily happen at the same time. Nuclear maturation, the meiotic resumption process, transforms prophase oocytes to metaphase II (MII) oocytes^(50)^. Following meiotic resumption, the nuclear membrane dissolves, which is called GV breakdown (GVBD). For the developmental ability and fertilization of oocytes, cytoplasmic maturation seems to be as important as nuclear maturation^(51)^. The first live baby born from IVM, was the result of oocytes picked up at different stages of the menstrual cycle and derived from an unstimulated patient with PCOS^(18)^. Overcoming failure in IVM urged fertility specialists to transfer an average of 6.3 embryos^(52)^. Söderström-Anttila et al.^(53)^ published an article on IVM from unstimulated patients with normal or PCOS ovaries and evaluated 239 cycles of immature oocyte retrievals without gonadotropin stimulation. Nine-hundred seventy-one immature oocytes from 122 IVF-IVM cycles were compared with 851 immature oocytes from 117 intracytoplasmic sperm injection (ICSI)-IVM cycles and found maturation and fertilization rates in IVF was 62.6% and 37.7% after IVF, whereas these rates were 53.9% and 69.3% after ICSI, respectively. The implantation rates and pregnancy rates were higher in the IVF-IVM group. They concluded that good pregnancy rates could be achieved in IVM cycles without gonadotropin stimulation and ICSI or IVF did not significantly change success rates^(53)^. Walls et al.^(54)^. studied IVF versus ICSI for fertilization in IVM cycles and compared 72 IVM-IVF cycles with 78 IVM-ICSI cycles and were able to follow up the embryos until the blastocyst stage. Blastocyst stage embryos were determined at higher rates in IVF-IVM than in ICSI-IVM, and the maturation rates were similar. It was concluded that IVM-IVF could be a viable method for obtaining good quality embryos and acceptable clinical pregnancy rates in PCOS-IVM cycles^(54)^. In order to increase the success rates in IVM cycles, priming with FSH or hCG has been recommended before oocyte retrieval^(55,56,57)^. Smith et al.^(58)^ were first to describe the 10 mm cut-off value for the size of the leading follicle in order to obtain immature oocytes during retrieval. Management of OHSS is a major concern of IVM because FSH-priming stimulation of the ovaries will not trigger the secondary or tertiary cohorts in patients with PCOS who prefer IVM as the treatment of choice. In some circumstances, early follicular aspiration at follicular size <14 mm and *in vitro *oocyte maturation as an adjunct to matured oocytes may be preferred to overcome OHSS, which is known as rescue IVM (rescue IVM is also used for patients converted from conventional IVF due to difficulty in follicular growth in stimulated cycles)^(59)^. Fadini et al.^(60)^ evaluated the effect of different modes of stimulation in IVM patients with normoovulatory ovaries and assessed 400 eligible women for the study. One hundred patients were treated without FSH priming, 100 were primed with hCG alone, 100 were primed with FSH, and finally, the last 100 patients were primed with both FSH and LH. The results of the study showed that FSH priming together with hCG priming had favorable outcomes compared with the other modalities. FSH priming or hCG priming alone makes no significant contribution to the clinical outcomes^(60)^. Contrarily, Mikkelsen and Lindenberg^(56)^ stated that priming with FSH alone in IVM cycles might improve the maturational potential and implantation rates of the embryos derived from immature oocytes. Reinblatt et al.^(61)^ studied the controversial issues in IVM and they categorized the problematic conditions under four areas, namely: 1) the benefits of gonadotropin use, 2) hCG priming and timing, 3) ideal endometrial preparation, and 4) luteal phase support. The authors concluded that prospective randomized and well-designed studies were necessary for both clinical applications and maturational processes of oocytes *in vitro*^(61)^. hCG priming may be substituted with recombinant LH in IVM cycles. Hreinsson et al.^(62)^ used recombinant LH instead of hCG for priming in a randomized trial and found that rec LH was as efficient as hCG in promoting the maturation processes of immature oocytes *in vitro*. However, rec FSH use did not seem to be patient friendly because of the costs when compared with hCG. A French group reported their clinical outcomes in 33 PCOS patients with 45 IVM cycles in 2005 and obtained 26.2% pregnancy rate and concluded that hCG primed IVM might be an alternative to conventional IVF^(63)^. Farhi et al.^(64)^ studied the use of OCP before IVM to decrease the laboratory overload related to IVM procedures and compared this approach with immediate-start IVM and concluded that pregnancy rates were similar and programming of IVM cycles was possible. Vitek et al.^(65)^ published an article on estrogen-suppressed IVM as a new and efficient IVM protocol and they evaluated the clinical and laboratory aspects of ES-IVM. This approach had similar outcomes to natural-cycle IVM or FSH-priming IVM and might eliminate the dependence on gonadotropins during IVM cycles^(65)^. This is one of the most controversial issues in IVM because early-onset estrogen, either alone as in this study, or in combination with FSH may suppress endogenous FSH and may have undesired effects on the oocyte maturation *in vitro.* Early-onset estrogen may influence synchronized GV oocyte retrievals, which is desired to overcome terminologic confusion^(26)^. Another interesting stimulation protocol for IVM is the use of letrozole for flare up of FSH for a short time while blocking the receptors reversibly. Rose studied letrozole use in IVM cycles and achieved successful pregnancies and ongoing pregnancies and deliveries^(66)^. Robertson et al.^(67)^ studied letrozole use in IVM in 5 patients and achieved 3 pregnancies, 2 of which delivered healthy infants (Hatırnaz et al, article under evaluation). The use of letrozole is also very important in patients with cancer seeking fertility preservation and for those who need emergency IVM with a random-start protocol. Albuz et al.^(68)^ studied cyclic AMP modulators added to pre IVM of bovine or mouse COCs and this was determined to increase COC cyclic AMP levels almost 100-fold. By this way, they simulated oocyte maturation physiology and named their method ‘simulated physiological oocyte maturation’ (SPOM). SPOM mimics the oocyte maturation *in vivo* and has benefits for oocyte IVM, which may be used in IVM protocols for better clinical outcomes^(68)^. Another important issue in IVM protocol is the timing and dosage of hCG priming and the time interval between hCG priming and oocyte retrieval. Endometrial thickness >8 mm together with a leading follicle <14 mm is preferred for the timing of hCG priming in IVM cycles^(69)^. A leading follicle size of <12 mm is accepted for obtaining GV oocytes in IVM cycles. The McGill group published an article related to the time interval after hCG and concluded that instead of 35 hours, a 38 hour interval until oocyte retrieval increased the chance of oocyte maturation and might influence the laboratory and clinical outcomes in cycles for IVM^(70)^. IVM has wide variety of indications and is open to novel treatment options. Combined with the advances in culture systems and more standardized protocols, IVM will take a greater place in IVF centers rather than as a neglected modality in the modern ART era.

### Oocyte pick-up (retrieval)

Bovine studies have shown that the diameters of aspiration needles and vacuum aspiration pressure during immature oocyte pick-up (OPU) by the transvaginal route have significant impact on the morphology of COCs and this morphology is involved in the developmental capacity and competence of bovine oocytes^(71)^.

The recommended pressure during human immature oocyte retrieval varies from 56 mm hemoglobin (Hg) to 180 mm Hg and aspiration needle diameter ranges from 16-20 gauge^(18,29,72,73,74)^. The cross-sectional area of a 17-gauge needle is 3.57 times wider than that of a 20-gauge needle. The recommended vacuum pressure for IVM OPU is 80-100 mm Hg. Lower pressure aspiration together with a 20-gauge aspiration needle may improve the developmental competence of oocytes derived from IVM cycles^(75)^. Techniques of OPU, total time for OPU, the use of a flushing medium during immature OPU, and the temperature regulations in the aspiration pump system are also important factors for the developmental potential and cytoplasmic maturation of oocytes* in vitro*.

### Laboratory procedures

Mammalian oocytes are dependent on the follicular environment for proper maturation. Oocytes and follicles have symbiosis-like interrelations because the follicle loses its competence when the oocyte ovulates from the follicle. In the meantime, oocyte development and meiotic resumption takes place in the follicular milieu after the LH peak. Removal of immature oocytes from the follicles blocks the completion of maturation processes and no well-developed culture environment will be sufficient to perfectly nourish and mature the oocytes derived from IVM. There have been more animal studies on IVM than human studies and information derived from large animal studies may illuminate the path of human oocyte IVM. The follicular conditions at the time of oocyte recovery and the oocyte chromatin distribution may have a great impact on the clinical outcomes in human IVM studies^(76)^. Chromatin condensation begins when the preantral follicles become early antral follicles in mammalian oocytes. The oocyte itself can determine its own fate. Oocyte-derived growth differentiation factor (GDF) 9 and bone morphogenetic protein (BMP) 15 have regulatory roles on the proliferation of granulosa cells, thus the oocyte has great potential to arrange its own environment for nuclear and cytoplasmic maturation^(77)^. The use of human and animal model studies together with advancing technologies may open new areas of IVM use in ART practice. It is important to know that the origin of the germ-line stem cells does not have to be ovarian but may be derived from bone marrow or peripheral blood, as reported by Edwards^(78)^. In an experimental study, the oocytes in the ovaries of mice were destroyed chemically and the oocytes removed and later injected with bone marrow cells and the chemically-depleted ovary resumed small antral follicle formations^(79)^. Adding matrix metallopeptidase to heat-stressed bovine oocytes during IVM has not been shown to improve the *in vitro *oocyte growth and even resulted in a detrimental effect^(80)^. The development of culture systems for animals provides valuable information to develop culture media for human oocyte IVM. For the monitoring of oocyte competence and maturation *in vitro*, a three-step culture system has been developed but the processes *in vivo* is accelerated in culture systems with optimizations for oocyte competence^(81)^. It is important to understand the cellular and molecular events that occur in the follicular environment coordinate both oocyte and somatic cell development. This course of events eventually raises the quality of the consistency of culture media used for *in vitro* oocyte maturation in humans. Oocyte-secreted factors, mainly GDF 9 and BMP 15, regulate the COCs and cumulus cell function and follicular granulosa cell functions *in vivo* and have a great impact on the quality of oocytes^(82,83)^. Understanding the intrafollicular environment and developmental mechanisms of oocytes *in vitro* will clarify the apoptotic processes that result in oocyte atresia or EFS, which may be overcome with IVM procedures^(47,48)^. The quality and consistency of the culture media used for *in vitro* oocyte maturation remains a dilemma in the era of extensive gonadotropin use. OHSS, which is a complication of IVF drugs, is a life-threatening condition and the risk is almost zero in IVM cycles, although it has been attempted to resolve the complication related to the drugs used in IVF with another drug instead of IVM. This is an important matter and few reports support the idea that IVM is useless in modern ART. The number of patients preferring IVM as the treatment of choice has reduced and financial resources for culture media development are restricted. Although there are a few publications opposing IVM^(22,23)^, there is an increasing number of publications supporting IVM in both clinical and ultrastructural points. Access to IVM culture media whenever required is not easy due to the reduced demand of centers for IVM, but in reality, all IVF laboratories need to learn and include IVM in their routine practice, rather than neglect it. Therefore, follicular fluids (FF) are co-cultured with culture media together with cumulus corona complex or hCG, and FSH in predetermined doses is added to the culture environment to support the development of immature oocytes. Such additions may influence the nuclear maturation but have no effect on cytoplasmic maturation^(84,85)^. Early embryonic development has many interactions and complex intracellular and extracellular correspondence. Studies have reported a number of physiologic changes in culture environments for remarkable oocyte growth and maturation *in vitro* although fully imitating *in vivo *conditions seems impossible. Adding FSH, LH, and human serum albumin to the culture medium of IVM means that there is much to do to develop well-standardized culture media^(86)^. IVM of human oocytes is a necessity for different ovarian pathologies. Investigating human oocytes for maturation problems has ethical issues and limitations. The major determinant of embryonic development is the quality of the oocyte, which is the GV oocyte in IVM. Two GV oocyte types have been recorded; one is the surrounded nucleolus (SN) and the other is the non-SN (NSN). SN GV oocytes have great developmental competence compared with NSN, which can be determined by heterochromatin staining around the nucleolus. Finding SN oocytes may improve the clinical outcomes of IVM cycles. Besides the low developmental potential or arrest of the NSN oocytes, there is also a relationship with the reduced expression of ribosomal proteins, especially cytoplasmic lattices, which can be used as morphologic markers^(87)^. Culture media used in in vitro oocyte maturation is the cornerstone of IVM cycles, but trials on the efficiency of the culture media and studies to develop much better cultures are unfortunately limited. Two IVM culture media are currently used in IVM practice and in one study immature oocytes derived from C-section patients were shared in Medicult and Sage culture media and the results were compared. The authors concluded that there was no difference between the media in respect of fertilization, cleavage, and blastocyst formation rates^(88)^. Filali et al.^(89)^ reported a retrospective study on the efficacy of (two culture media-199 and IVM-Medicult). Both media showed similar outcomes concerning oocyte maturation, fertilization, embryonic development, and clinical pregnancy rates^(89)^. For immature oocytes derived from stimulated IVF patients, commercially available IVM culture systems may not be enough for the maturation of GV and MI oocytes^(90)^. IVM culture media need some additions for oocyte IVM , mainly autologous serum of the patient and FSH and LH added as stock solutions. Lin et al.^(91) ^and Goud et al.^(92) ^studied adding growth factors [Epidermal growth factor (EGF), insulin-like growth factor-1 (IGF-1), activin, transforming growth factor-beta or granulosa cell co-culture) in standard IVM medium for immature mouse oocytes and concluded that the addition of growth factors did not improve the clinical outcomes^(91,92)^. Contrary to the above publication, Jahromi et al.^(93)^ reported that the addition of granulosa cells in human oocyte IVM medium as a co-culture might improve the developmental competence and maturation of oocytes. Optimized IVM procedures should be studied extensively in well-designed human clinical trials instead of animal models. Animal studies have shown correct imprinted DNA methylation establishment in oocytes but such a conclusion cannot be reached in human IVM. Epigenetic analyses from babies born from IVM treatment will clarify the epigenetic safety of the procedure in humans^(94)^. hCG priming IVM-derived oocytes are correlated with their maturation time and those oocytes reached MII in a shorter time than other GV oocytes, and had higher developmental competence and better success rates. This study showed that *in vitro* culture systems favored nuclear maturation in human oocytes but cytoplasmic competence needed further studies to be improved^(95)^. IVM may have some undesired effects on the chromosomal alignments and spindle structure of the immature oocytes within the culture environment. In a study, 304 oocytes from 101 women and spindle configurations were detected by using PolScope and evaluated with immunohistochemical staining for alpha tubulin and chromatin. The authors concluded that supplementing the culture media might improve the maturation rate but not indicate the presence of spindles and chromosomal alignment^(96)^. The culture media and the plastic materials used in laboratories are important in oocyte IVM. Bisphenol-A (BPA) may, in a dose-related manner, decrease the MII transformation of GV oocytes and increase the rate of oocyte degeneration in the laboratory. Chromosomal alignment and bipolar spindle formation decreases as MII oocytes come into contact with higher BPA concentrations^(97)^. Elizur et al.^(98)^ evaluated 15 women to evaluate the corpus luteum formation potential of minimum-sized follicles during hCG priming IVM cycles and measured estrogen and progesterone levels 5-7 days after oocyte retrieval and antral follicles were recorded. They discovered a new cohort of follicles during their study, which supported the studies of Baerwald et al.^(99)^. The occurrence of fertilization failure in IVM and IVF may be attributed to the short interval of hCG triggering and ICSI and the lengthening of this interval may overcome the risk of FF in IVM cycles^(100)^. Nuclear and cytoplasmic maturity are sequential but independent processes in the maturation of oocytes and culture-related conditions may alter the course of maturation processes, mainly cytoplasmic maturation, and eventually may result in abnormalities or in increased aneuploidy rates. For this reason, oocyte-donor immature oocyte-derived embryos from 11 patients were compared with embryos for PGD-sex selection. The study revealed that preimplantation genetics or prenatal chromosome studies should be recommended to patients preferring IVM as the treatment of choice^(101)^. An important study on the chromosomal abnormality rates in IVM cycles was conducted in McGill University and 6 IVM cycles and 30 IVF cycles for fluorescence *in situ* hybridization (FISH) analysis were compared and the results showed that the aneuploidy rates in both groups were similar. The aneuploidy rate in IVM patients with a maturation interval of 48 hours had significantly higher aneuploidy rates compared with 24-hour interval matured oocytes^(102)^. Real-time continuous embryonic follow-up by time-lapse incubation in IVM cycles of patients with PCOS was studied revealing an increase in the early embryonic arrest but the morphokinetic changes during embryonic development were not altered. Multinucleation at the two-cell and four-cell stage and uneven blastomeres at the two-cell stage were higher in PCOS-IVM cycles. Embryo arrest at day 3 to day 4 transition was seen to be higher in PCOS-IVM patients^(103)^. Improved culture systems and optimized protocols in PCO-IVM and PCOS-IVM patients led to good blastocyst formation, improved implantation and pregnancy rates in single-embryo transfer. One hundred to 150 IU FSH started at day 3 of the cycle for 3 days plus hCG priming when the leading follicle reached 10-12 mm and estrogen added to the protocol on the day of oocyte retrieval when the endometrium was 6 mm. Eight hundred forty-four oocytes from 66 patients were collected and 588 oocytes were matured in vitro, 420 oocytes were fertilized, and 175 embryos reached blastocyst stage. Sixty-two good grade blastocyst embryos were transferred as single-embryo transfer and 28 live births were achieved. The authors concluded that optimized IVM protocols might result in good laboratory and clinical outcomes^(104)^. Early estrogenic supplementation on day 3 in both FSH and LH priming IVM cycles of 159 PCOS patients for optimum clinical outcomes revealed that homogenous immature oocyte retrievals were achieved in such cycles and single-embryo transfer was a feasible option in IVM cycles and prevented multifetal gestations^(26)^.

### Ultrastructural changes *in in vitro* matured oocytes

EGF and IGF-1 were found to augment the spontaneous maturation of oocytes *in vitro*^(105,106)^. After publications of mouse FF-meiosis activating sterol (MAS), a trial was held to induce *in vitro* oocyte maturation by the addition of MAS in human oocytes, and it was seen that FF-MAS positively influenced human oocyte morphokinetics and nuclear maturation^(107)^. The supplementation of forklosin, an adenylate cyclase activator, and cilostamide, a phosphodiesterase inhibitors specific inhibitor, were added to the maturation culture media of immature oocytes and these agents were found to be influential in maturation processes and meiotic resumption^(108)^. Different maturation stages of oocytes have different morphokinetic characteristics during IVM. Cumulus cells surrounding abnormal oocytes carry high apoptotic potential, and abnormal oocytes had degenerated cellular structures in ultrastructural studies. Electron microscopic (EM) studies revealed that microvilli were rare around GV oocytes, the zona pellucida is thin and loose outside. Following GVBD, microvilli surrounding MI oocytes are common and large in size. EM of MII oocytes has shown mitochondrial degeneration and diminished cristae in the mitochondria (M) together with an increased amount of apoptotic activity in cumulus cells^(109)^. In one study, 204 immature oocytes were evaluated for ultrastructural changes and 101 GV oocytes were compared with 103 MI oocytes. Maturation rates were higher in MI oocytes and immature oocytes showed large M-vesicle complexes VCs. Transmission EM findings of MII oocytes were dense fibrillary zona pellucida, uniform perivitelline space, a continuous oolemma and regularly distributed microvilli. The ultrastructure of GV oocytes from IVM have similarities with MII oocytes, but the most pathognomonic finding of GV oocytes is numerous large M-VCs. The authors concluded that immature human oocytes from IVM at different stages of development showed minimal cytoplasmic alterations in EM studies^(110)^. *In vitro* matured oocytes were evaluated ultrastructurally before and after vitrification and vitrified thawed oocytes were compared with previtrified oocytes concerning ultrastructural changes including M-smooth endoplasmic reticulum M-vs, the number of cortical granules, the integrity of oolemma and microvilli, vacuolization, and surrounding zona. The authors concluded that the ultrastructure of matured oocytes from IVM cycles had similar cytoarchitecture in both vitrified-warmed and previtrified oocytes in EM studies^(111)^. Dal Canto et al.^(112)^ studied the morphokinetics of embryos derived from *in vitro* matured oocytes from hCG-primed IVM cycles. Oocytes derived from 8-12 mm follicles were categorized according to the cumulus expansion and expanded cumulus oocytes were evaluated as MII and incubated for 6 hours, whereas others were accepted as GV oocytes and incubated for 30 hours. The authors concluded that morphokinetic behavior of both mature and GV oocytes from IVM cycles were comparable and it was suggested that only minimal differences were present in GV and MII oocytes from IVM cycles^(112)^. Embryos derived from GV oocyte maturation in stimulated IVF cycles had a high arrest rates and multinucleation rates but meiotic resumption happened normally. The aneuploidy rates were higher among these embryos determined by blastomere biopsy and FISH analysis^(113)^. The impact of oocyte IVM on the distribution of M was studied in China. Two hundred eighty-four immature oocytes derived from stimulated IVF cycles were evaluated and 140 were fixed. Other oocytes were prepared for IVM before the fixation process. All 21 oocytes matured *in vivo* were fixed directly and stained to visualize the M. The mitochondrial distribution was observed using confocal microscopy. Three types of mitochondrial distribution pattern were observed; peripheral, semiperipheral, and evenly diffused. Pre IVM oocytes showed high peripheral distribution and post IVM oocytes showed evenly-diffused M. The M of *in vivo* mature oocytes showed more central localization. The authors concluded that this might explain the diminished developmental competence of the IVM oocytes^(114)^. Similar outcomes were reported by Takahashi et al.^(115)^. Melatonin could induce meiotic maturation in bovine and porcine immature oocytes. This investigation illuminated the study of low- concentration melatonin use in human oocytes and 1 nM dose was found to be optimal for human GV and MI oocytes and to have a positive influence on nuclear maturation during rescue IVM^(116)^. Why do *in vitro* matured oocyte-derived embryos have low implantation potential? IVM and *in vivo*-matured (IVO) oocytes derived from pseudopregnant mice were compared, and after 5 days, the implanted blastocysts were removed from the mouse uterine horns and the uterine horns were analyzed for mRNAs, some growth factors, progesterone receptors, and homeobox A10. The maturation rates of GV oocytes were high but the implantation and fertilization rates were quite low compared with IVO and all mRNAs derived from IVM derived embryo horns were significantly diminished compared with IVO. The authors concluded that implantation-related mRNAs were diminished and thus the developmental competence of IVM-derived embryos was lower than IVO^(117)^. The presence of double-strand DNA breaks were found in immature oocytes and DNA integrity was an integral part of meiotic resumption in IVM^(118)^. Reduced oxygen concentrations in the laboratory environment for the development of immature oocytes and embryos is an important factor that can be measured by the expression patterns of glucose metabolism genes^(119)^. PGS or PGD for some genetic problems may be applied in IVM embryos that have reached the blastocyst stage and the first healthy baby born from PGD for chromosomal translocation was reported in an IVM cycle^(120)^. Practicing PGD in IVM may eliminate developmentally-incompetent and aneuploid embryos, which eventually may improve the implantation and pregnancy rates with healthy deliveries.

### Vitrification in *in vitro* maturation

Cryopreservation of *in vitro* matured oocytes is a new dimension in assisted reproduction. Cryoprotectants may have oocyte meiotic spindle damage and may alter mitochondrial function and integrity during vitrification. *In vitro*-matured oocytes from donors have shown that spindle and chromosomes were not affected by vitrification solutions^(121)^. There are a few alternative kits for vitrification. An Italian group studied the use of Cryotop vitrification in IVM GV oocytes and compared them with GV oocytes and MII oocytes from fresh cycles. Light microscopy and phase contrast microscopy results showed no significant changes in the oolemma and cytoplasm. The ultrastructural features of GV oocytes were preserved during Cryotop vitrification and the authors concluded that the GV stage seemed more suitable for vitrification than MII oocytes^(122)^. To determine the strength of GV oocytes, 184 immature oocytes were divided into two groups and 100 MII oocytes were vitrified for 24-48 hours after IVM and another 84 GV oocytes were vitrified directly and *in vitro* matured after thawing. The survival of the thawed oocytes and thawed and *in vitro*-matured oocytes were similar but the maturation rate was higher in the group that was matured first and vitrified later. The authors concluded that IVM maturation was more efficient when performed before GV oocyte vitrification^(123)^. Day 3 vitrified-warmed embryos from *in vitro* matured oocytes have the same potential to reach the blastocyst stage compared with fresh embryos. However, vitrified oocyte-derived embryonic transition to cleavage stage embryos, namely day 3, was low, and the authors concluded that *in vitro*-matured oocyte-derived embryonic development before day 3 was diminished but after genomic activation; embryos that pass beyond day 3 to day 5 were not affected by vitrification^(124)^. During maturation, oocytes use Ca^2+^ for many physiologic processes and in mouse studies, vitirification solutions including dimethyl sulfoxide caused temporary rises in Ca^2+^ concentrations. CP also increases intracellular Ca^2+^ concentrations. The same physiologic principles may be used in human oocytes and GV oocytes were randomized into five groups. G1; GV oocytes matured by IVM, G2; vitrified at GV stage, G3; GV oocytes matured by IVM and then vitrified, G4; human oocyte IVM through the intracellular oscillations, and G5; GV oocytes exposed to ionomycin and IVM until MII. The authors concluded that osmotic shock from the vitrification solutions might have influenced the maturation capacity of IVM oocytes^(125)^. Vitrification under 196 °C does not mean that the cryoenvironment is aseptic; therefore, protection of embryos or oocytes is mandatory and a carrier and storage system that separates the gametes and embryos from others is recommended for laboratories^(126)^.

### *In vitro* maturationin fertility preservation

Cancer and fertility preservation in young people is an important medical concern and needs to have strategies developed. For fertility preservation in patients with cancer, a multidisciplinary team including specialists in gynecological oncology, general surgery, oncologists, assisted reproductive technology team, clinical embryologists, and genetic specialists is necessary. Ovarian tissue freezing, immature oocyte harvesting from ovarian tissue specimens, and oocyte maturation *in vitro *become key troubleshooting factors in patients with cancer, especially if urgent cancer treatment is ahead^(127)^. Improved cancer treatment outcomes in young males and females have led physicians to fertility preservation measures to avoid major sequelae of rigorous cancer treatments. Sperm and oocyte cryopreservation, testicular and ovarian tissue freezing, and cryopreservation of embryos derived from IVM oocytes are great challenges for fertility preservation. However, some technical, legal, and ethical concerns have yet to be clarified such as informed consent from patients who are minors, legal parentage, and medical negligence^(128)^. Immediate IVM was preferred in a 27-year-old woman who presented for fertility preservation prior to pelvic radiotherapy and had a laparoscopic radical hysterectomy for cervical carcinoma. Due to the risk of vaginal dissemination risk of the disease, the ovaries were removed and oocytes were removed from the ovariectomy specimen by *ex vivo* aspiration and then 22 oocytes were matured *in vitro* and 15 oocytes were matured to MII oocytes in 24 hours and vitrified. The remaining oocytes were followed up for a further 24 hours and 7 more oocytes reached MII phase and were vitrified as a second round. This is a good modality of fertility preservation in patients with cancer who are short of time because of radiotherapy and chemotherapy^(129)^. A Canadian group evaluated 41 women with cancer who had undergone IVF treatment and compared them with 48 women as a control group. They found that younger women with malignancies maintained their ovarian reserve, responded to gonadotropins well, and oocyte retrieval and maturation rates were unchanged, but the same recommendations cannot be made for spermatogenesis^(130)^. The same group studied patients with breast cancer (n=87), hematologic malignancies (n=16), and gynecologic or abdominal malignancies (n=9) who were treated with IVM and compared them with 79 infertile controls. Ovarian reserve and maturity rates were found to be similar in malignancies other than breast cancer, and patients with breast cancer treated with IVM had fewer oocytes retrieved^(131)^. Fadini et al.^(132)^ reported a patient with ovarian cancer who was treated conservatively and oocytes were recovered from antral follicles, matured *in vitro*, and then the developed embryo was vitrified and later warmed; a 2-cell embryo was transferred but failed to achieve pregnancy. Ovarian stimulation in patients with breast cancer is almost impossible, thus unstimulated IVM has become a valuable option^(133)^. Oktay et al.^(134) ^studied the use of IVM as a complementary treatment for 32 patients with breast cancer and 464 oocytes were retrieved, of which 274 were matured. Immature oocytes were matured in IVM culture media and a total of 399 oocytes were matured; fertilization by IVM was higher than spontaneous maturation. Thus, IVM is a useful strategy for obtaining mature oocytes for fertility preservation^(134)^. IVM has found a new area of use in oophorectomy specimens and immature oocytes are recovered and cryopreserved, and babies delivered from those oocytes have been reported. An average 14 oocytes from each of 34 patients were retrieved with an overall maturation in vitro rate of 36%. Although most patients preferred oocyte vitrification, 8 patients preferred embryo freezing and 1 patient preferred to have embryo transfer and ongoing pregnancy was achieved after warmed embryo transfer^(135,136)^. Oocytes can be retrieved from postpubertal female children at risk of premature ovarian failure due to Turner syndrome or cancer; retrieved oocytes have been matured and cryopreserved from girls who accepted fertility preservation and all the required procedures were well tolerated^(137)^. Fertility preservation is not restricted to patients cancer, it can also be used in other medical conditions^(138)^. Indications of fertility preservation other than cancer are listed below:

- Premature ovarian failure

- Chromosomal and genetic abnormalities (Turner syndrome, 47, XXX, Fragile XGALT enzyme or FSH receptor mutation)

- Autoimmune diseases (thyroid, polyglandular, multiple endocrine)

- Environmental factors (malaria, varicella, Shigella may cause POF)

- Surgical menopause (benign ovarian disease, prophylactic oophorectomy)

- Cytotoxic agents for hematologic and autoimmune diseases

- Postponed fertility.

### Obstetric and perinatal outcomes in *in vitro* maturationin

The first baby born from immature human oocytes harvested from unstimulated ovaries from a gonadectomy patient and used in donor oocyte program was reported by Trounson et al.^(18)^. Cha et al.^(52)^ also reported clinical pregnancies and deliveries from IVM oocyte-derived embryos in 64 patients with PCOS. Twenty-three of 85 ET cycles of 64 patients with PCOS (27% pregnancy rate) resulted in pregnancy. Seventeen patients delivered 20 normal-appearing infants^(53)^. Söderström-Anttila et al.^(139)^ studied the obstetric and perinatal outcomes of children born from IVM cycles. IVM born babies were followed up carefully because this ART technique has rarely been applied. Forty-three women who delivered 40 singleton babies and three sets of twins were followed up for 2 years and the results showed that both perinatal-obstetric outcomes and development of the children in the two years’ follow-up were normal^(139)^. Early studies of IVM revealed embryo transfers on day 2-3, but recently, blastocyst transfers achieved and pregnancy outcomes related to blastocyst transfer have been published. From 106 hCG-priming IVM cycles of 82 patients, blastocyst transfer was achieved and the implantation rate was 26.8% and the pregnancy rate was 51.9%, which is favorable. Fifty-five cycles with blastocyst transfer resulted in clinical pregnancies. Forty-three women delivered 33 female and 24 male babies. The results were compared with cleavage-stage embryo transfer and the clinical pregnancy rate was found significantly higher in blastocyst transfer^(140)^. However, the rate of miscarriage among IVM cycles was higher as compared with IVF and ICSI cycles. This may be attributed to PCOS itself rather than IVM treatment^(141)^. In a study held in Italy, 196 babies (153 singletons and 43 twins) born from IVM treatments were evaluated for obstetric and perinatal outcomes. Among the twin pregnancies, one fetus was diagnosed with Down syndrome and  aborted. Obstetric and perinatal outcomes were compared in detail with ICSI outcomes of the control group and the authors concluded that the outcomes were comparable including the major and minor abnormalities^(142)^. An interesting study from Japan reported imprinting genes and epigenetic factors related to IVM babies. EM studies were performed on different stages of IVM oocytes for oxygen consumption and blood from umbilical cords of babies born from IVM treatments. Neither oxygen consumption of oocytes nor imprinting gene defects from babies delivered were found in IVM cycles and the authors concluded that IVM was not related to imprinting gene disorders^(143)^. Another group from Germany studied the DNA methylation pattern (epigenetic role) in children born from IVM-ICSI and compared 11 IVM-ICSI babies with 19 controls. Chorionic villus sampling and cord-blood sampling were used for the evaluation and their results showed that no significant differences were found in either group. With regards epigenetics, the frequency of such imprinting defects or expressions is quite low^(144)^.

### Concluding remarks

- The course of IVM is progressing slowly and has a long way to go.

- IVM seems to remain an alternative option until standardized terminology and stimulation protocols are in place.

- The best IVM program may be FSH-hCG priming, yielding 100% GV oocytes with favorable clinical outcomes.

- Nuclear maturation and cytoplasmic maturation are not concordant and cytoplasmic maturation needs to be investigated extensively.

- Embryonic arrest seems more prevalent within the first three days but embryos beyond day 3 are more competent.

- Epigenetic changes in IVM are not significant and not more than changes in conventional IVF.

- IVM can be applied to all indications in which conventional IVF is applied.

- Enrichment of culture media for cytoplasmic maturity may increase the clinical outcomes in IVM cycles.

- For fertility preservation, IVM seems a remarkable option.

- PGS/PGD can be easily performed from the embryos of IVM cycles.

- Embryos derived from IVM oocytes are more susceptible to cryopreservation than GV oocytes.

The enrichment of culture media, standardization of the stimulation protocols and management of cytoplasmic maturity are strongly recommended for improved IVM cycles. Future fertility preservation and young age malignancies draw attention on IVM and as a conclusion, increasing the experience of IVM is recommended for all IVF laboratories, instead of neglecting it.
